# Comparing *Mycobacterium tuberculosis* transmission reconstruction models from whole genome sequence data

**DOI:** 10.1017/S0950268823000900

**Published:** 2023-06-09

**Authors:** Benjamin Sobkowiak, Kamila Romanowski, Inna Sekirov, Jennifer L. Gardy, James C. Johnston

**Affiliations:** 1 Division of Respiratory Medicine, University of British Columbia, Vancouver, BC, Canada; 2 British Columbia Centre for Disease Control, Vancouver, BC, Canada; 3 Department of Medicine, University of British Columbia, Vancouver, BC, Canada; 4 Department of Pathology and Laboratory Medicine, University of British Columbia, Vancouver, BC, Canada; 5 Bill and Melinda Gates Foundation, Seattle, WA, USA

**Keywords:** Bacterial infections, tuberculosis (TB), transmission, infectious disease epidemiology, bioinformatics

## Abstract

Genomic epidemiology is routinely used worldwide to interrogate infectious disease dynamics. Multiple computational tools exist that reconstruct transmission networks by coupling genomic data with epidemiological models. Resulting inferences can improve our understanding of pathogen transmission dynamics, and yet the performance of these tools has not been evaluated for tuberculosis (TB), a disease process with complex epidemiology including variable latency and within-host heterogeneity. Here, we performed a systematic comparison of six publicly available transmission reconstruction models, evaluating their accuracy when predicting transmission events in simulated and real-world *Mycobacterium tuberculosis* outbreaks. We observed variability in the number of transmission links that were predicted with high probability (*P* ≥ 0.5) and low accuracy of these predictions against known transmission in simulated outbreaks. We also found a low proportion of epidemiologically supported case–contact pairs were identified in our real-world TB clusters. The specificity of all models was high, and a relatively high proportion of the total transmission events predicted by some models were true links, notably with TransPhylo, Outbreaker2, and Phybreak. Our findings may inform the choice of tools in TB transmission analyses and underscore the need for caution when interpreting transmission networks produced using probabilistic approaches.

## Introduction

Tuberculosis (TB), predominately caused by infection with *Mycobacterium tuberculosis* (*Mtb*), remains a major global public health concern, with an estimated 10.6 million people developing disease and around 1.5 million deaths in 2021 [1]. Accelerating the reduction in TB burden to meet the World Health Organization’s TB elimination goals will require improvements in our understanding of *Mtb* transmission dynamics within different population settings. This information is critical to informing management of local epidemiology and developing data-driven prevention and control strategies based on an understanding of the clinical, social network, and environmental factors that drive onward transmission.

At the individual and population levels, identifying linked cases and broader transmission clusters has traditionally been done through a combination of DNA fingerprinting techniques and field-based epidemiological methods, such as contact tracing [2]. While this approach can be effective in well-resourced settings with low TB burden, it can be prohibitively labor-intensive in high transmission settings with limited laboratory and field epidemiology capacity, as well as inherently subjective when tracing relies on interviewee recall, which can be particularly problematic for an illness with such a prolonged infectious period. This can limit epidemiologists’ ability to accurately reconstruct full transmission histories.

Whole genome sequencing (WGS) has enabled finer-scale resolution of *Mtb* transmission events. However, relating genomic variation to direct transmission can be complex when clinical and epidemiological information, such as symptom onset date, infectious period, and contact history, are incomplete. Simplistic methods, such as setting a threshold of single nucleotide polymorphism (SNP) differences to define recent transmission, have been applied successfully in multiple settings to draw insights into transmission dynamics [3, 4]. This, though, often lacks sufficient resolution to determine the direction and timing of individual transmission events [5, 6], and is further complicated by the lack of consensus on appropriate SNP thresholds [3, 7–9]. A more sophisticated approach to transmission reconstruction using genomic data relies on phylogenetic trees, named phylodynamics [10]. This characterises macro-scale network patterns [11] and can be used to accurately estimate transmission events and timings for some pathogens, such as RNA viruses [12, 13]. Multiple factors can complicate the use of phylodynamics in TB transmission reconstruction, including within-host evolution with long and variable latency periods and low sequence divergence due to a relatively low mutation rate [12, 14, 15]. Indeed, it has been previously suggested that sequencing data can provide limited information about person-to-person transmission in *Mtb* [13].

Several computational tools have been developed that combine genomic variation with an underlying epidemiological model (e.g., susceptible-infected-recovered (SIR) [6, 16] or stochastic branching process [15, 17]) to estimate the probability of individual-level transmission events from genomic data. These tools mainly employ a Bayesian Markov Chain Monte Carlo (MCMC) framework to account for the high dimensionality and computational complexity of the resulting models, as well as incorporating other epidemiologically derived parameters. Recently, multiple computational approaches were evaluated for reconstructing transmission in foot-and-mouth outbreaks [18], and while many of these tools have been used in *Mtb* transmission studies [19–21], their performance has not been systematically compared in TB. In this study, we evaluate and compare six model-based transmission inference approaches for reconstructing transmission networks using genomic data from real-world *Mtb* isolates collected in British Columbia (BC), Canada, and simulated TB outbreaks.

## Methods

### Mycobacterium tuberculosis isolates from British Columbia

Study data were collected in BC, a low TB burden region with a population of 5 million people and a TB incidence of six cases per 100,000 population [22, 23]. *Mtb* samples were obtained from the Public Health Laboratory (PHL) of the BC Centre for Disease Control (BCCDC). From 2,915 culture-positive TB cases diagnosed between 2005 and 2014, genomic DNA was extracted from 2,290 isolates, one sequence per person, and analysed using MIRU-VNTR genotyping. Sample preparation, DNA extraction, and genotyping methods are described elsewhere [24]. Ethics were obtained from the University of BC (certificate H12-00910) and informed consent for participation in the study was not required, as determined by institutional REB review.

WGS was performed at the BC Genome Sciences Centre on the Illumina HiSeq platform on all isolates with a shared MIRU-VNTR genotyping pattern. This resulted in 1,014 high-quality whole genome *Mtb* sequences of 125 bp paired-end reads, with an average depth of coverage of >100×. Reads were mapped to the H37Rv reference strain (NC_000962.3) using BWA *mem* [25]. Variant calling was conducted using GATK [26] programs *HaplotypeCaller* and *GenetypeGVCFs*, with SNPs used in the subsequent analysis. Low confidence variants (phred quality score Q < 20, read depth DP < 5) and sites with a missing call in >10% of isolates were removed. Heterozygous sites were called as the consensus allele if ≥80% of mapped reads corresponded at these positions or an ambiguous call ‘N’. Variants in repetitive regions, in PE/PPE genes, and at known resistance-conferring genes were removed from subsequent analysis.

Twelve putative transmission clusters were identified by grouping isolates with a shared MIRU-VNTR pattern where contract tracing data linked at least two cases in the cluster, categorised as either small (four clusters between five and nine isolates) or large (eight clusters with ≥10 isolates). Timed phylogenies were produced as a maximum clade credibility (MCC) tree for each MIRU-VNTR cluster separately with BEAST2 v2.6.3 [27], calibrated at the tips by collection date. Full details of the phylogenetic tree construction pipeline can be found in the Supplementary Methods. For analysis of multiple phylogenetic trees simultaneously with TransPhylo, a random selection of 50 trees was drawn from the posterior selection of 10,000 trees, discarding the 50% burn-in.

### Transmission network reconstruction models

We tested six tools for reconstructing transmission networks: seqTrack [28], TransPhylo [15], Outbreaker2 [29], Phybreak [17], SCOTTI [30], and BEASTLIER [16]. Table 1 shows the specific model features and input data types. An extension of TransPhylo to allow for parameter sharing and transmission inference from multiple input phylogenies was also tested, which we refer to here as TransPhyloMT [31]. In addition, we applied BEASTLIER using two approaches; jointly inferring the phylogenetic and transmission tree and fixing the phylogeny using the MCC timed tree for each cluster, only estimating the transmission tree (referred to here as BEASTLIER_fixed). We also attempted to run BadTrIP [32], though it failed to converge for most clusters. All approaches use genomic data, either from a multiple sequence alignment directly or from a timed phylogenetic tree indirectly (Table 1), and sampling dates. Priors chosen for each method reflect those previously used in transmission analysis of this TB population and in other settings with effective active case-finding strategies [15, 33, 34]. Full model-specific inputs and prior parameter estimates are found in the Supplementary Methods.

**Table 1.** The transmission network reconstruction tools evaluated in this study, detailing the epidemiological features and input data type for each tested approach

Transmission reconstruction model	Input data type	Prior generation time distribution	Prior mutation rate estimate	Can use spatial data	Can use contact-tracing data	Within-host evolution	Unsampled hosts	Estimates infection times
BEASTLIER	SNP alignment	Yes	Yes	Yes	No	Yes	No	Yes
Outbreaker2	SNP alignment	Yes	Yes	No	Yes	Yes	Yes	Yes
Phybreak	SNP alignment	Yes	Yes	No	No	Yes	No	Yes
SCOTTI	SNP alignment	No	Yes	No	No	Yes	Yes	No
seqTrack	SNP distance matrix	No	Yes	Yes	No	No	No	No
TransPhylo	Timed phylogenetic tree(s)	Yes	Yes	No	No	Yes	Yes	Yes

### Simulated transmission clusters

We simulated a total of 20 TB outbreak clusters using two simulation approaches. Ten clusters were using the ‘sim.phybreak’ function in Phybreak [17], which produces simulated sequence data and transmission networks, including the timing and direction of transmission. Ten outbreaks were simulated using the TransPhylo ‘simulateOutbreak’ function to obtain simulated transmission trees. The underlying phylogeny was extracted with the branch lengths converted to substitutions at a rate of 0.5 SNPs per genome per year, and sequence data simulated along the phylogeny using SeqGen [35]. Simulated clusters were produced with parameters to reflect the epidemiology and sampling strategy in the real-world *Mtb* clusters used in this study, including accounting for unsampled hosts. Full details of the simulation methods and parameters are presented in the Supplementary Methods. Simulated genome sequences and sampling dates of observed hosts were used as input for each tested method. Predicted transmission events were compared against the known transmission network to evaluate model sensitivity (the proportion of the true transmission links correctly identified), specificity (pairs that were correctly predicted not to be linked as a proportion of all true negative links), and the positive predictive value (PPV; the correct transmission links as a proportion of the total inferred transmission links (true positives + false positives)). Additionally, simulated transmission networks included information on who-infected-whom, so measures were also calculated when accounting for the correct direction of transmission.

### Model performance of Mycobacterium tuberculosis from British Columbia

In the absence of a gold standard for confirmed transmission in our real *Mtb* clusters, the performance of each tool was evaluated by determining the number of epidemiologically linked case–contact pairs that were correctly identified by each model (Supplementary Table S2). Case–contact pairs were characterised as two hosts found in the same MIRU-VNTR cluster where one host has named the other as a contact in contact-tracing questionnaires conducted after TB diagnosis. Correct predictions were considered as an inferred link between a known case–contact pair identified with a probability of ≥0.5. Where multiple donor hosts were predicted for a recipient strain, the highest probability link was considered.

Additionally, we compared posterior estimates of transmission parameters to evaluate the credibility of the predicted links. Previous work has reported that a signal of direct transmission in *Mtb* is low divergence between sequences, with most transmission pairs differing by fewer than five SNPs [3, 5, 36]. While there may be some cases of sequences differing by more than five SNPs in direct transmission events, we assumed that most transmission will be between isolates with few SNP differences and an abundance of links between highly diverged sequences may indicate erroneous inferences. Four of the tested approaches, excluding seqTrack and SCOTTI, inferred an infection time for each host. From these estimates, we calculated transmission intervals as the time between donor and recipient host infection.

## Results

### Simulated TB transmission clusters

**Figure 1.**
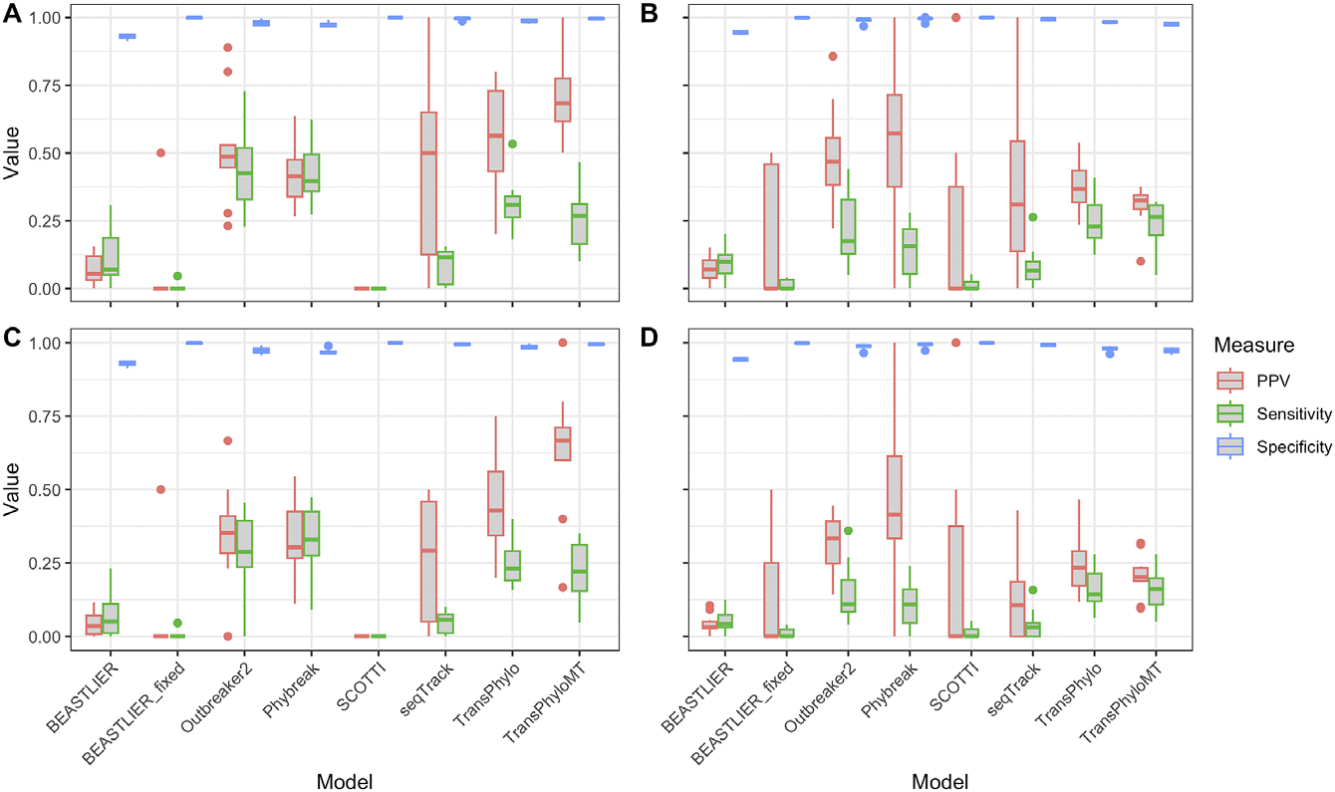
Boxplots showing the sensitivity (green), specificity (blue), and PPV (red) of each transmission reconstruction model for predicting known transmission events in 20 simulated tuberculosis outbreaks. Links with a probability of ≥0.5 are considered. The results when transmission links between pairs in any direction are shown for Phybreak simulations in (a) and TransPhylo+SeqGen simulations in (b). The results when transmission links are predicted with the correct donor–recipient direction are shown for Phybreak simulations in (c) and TransPhylo+SeqGen simulations in (d).

Figure 1 shows the sensitivity, specificity, and PPV of each tested approach from 20 simulated *Mtb* clusters, 10 each from two different simulation approaches. Transmission links with a probability of ≥0.5 were considered. Figure 1a,b show the results irrespective of the direction of the inferred transmission (i.e., if host *i* transmitted to host *j* in the true transmission network, a prediction of i -> j or j -> i would be scored as correct), and Figure 1c,d show the results when only links with the correct direction of transmission are predicted.

While there were differences in the overall performance of all tested methods between the two different simulation approaches, there were similarities in the relative performance of the tools across all simulations. Outbreaker2, Phybreak, TransPhylo, and TransPhyloMT correctly predicted the highest proportion of true transmission events in all simulations. In simulated outbreaks produced using Phybreak, Outbreaker2 achieved the highest sensitivity when not considering the direction of the transmission link (median 0.425, IQR 0.33–0.52), followed by Phybreak (sensitivity 0.40, IQR 0.36–0.49) (Figure 1a). For TransPhylo+SeqGen simulated outbreaks, TransPhyloMT had the highest sensitivity (median 0.26, IQR 0.2–0.31), followed by TransPhylo (median 0.23, IQR 0.19–0.31) (Figure 1b). BEASTLIER, BEASTLIER_fixed, seqTrack, and SCOTTI correctly predicted very few links between transmission pairs in all simulated clusters, resulting in low sensitivity when using these models (Supplementary Table S1). When the direction of transmission between host pairs was considered, the sensitivity of all models reduced compared to the sensitivity in predicting transmission links in either direction (Figure 1c,d). The relative accuracy of the tested models remained similar when predicting the direction of transmission, with the highest sensitivity in Phybreak simulations now achieved in Phybreak (median 0.33, IQR 0.275–0.42) and by TransPhyloMT in the TransPhylo+SeqGen simulated outbreaks (median 0.16, IQR 0.11–0.20).

There were marked differences in the PPV of each tested model. When not accounting for the direction of transmission, TransPhyloMT achieved the highest PPV (median 0.68, IQR 0.62–0.78) in the simulated outbreaks produced using Phybreak, and Phybreak had the highest PPV in outbreaks simulated using TransPhylo+SeqGen (median 0.57, IQR 0.37–0.71). The same models had the highest PPV scores when the direction of transmission was considered. We found the specificity of all tested models to be high in the simulated clusters. Only BEASTLIER had a median specificity of lower than 0.95 in all simulations, with and without considering the direction of transmission, due to the high number of transmission links predicted with this tool with a probability of ≥0.5 (Supplementary Table S1).

### Mycobacterium tuberculosis clusters from British Columbia, Canada

**Figure 2.**
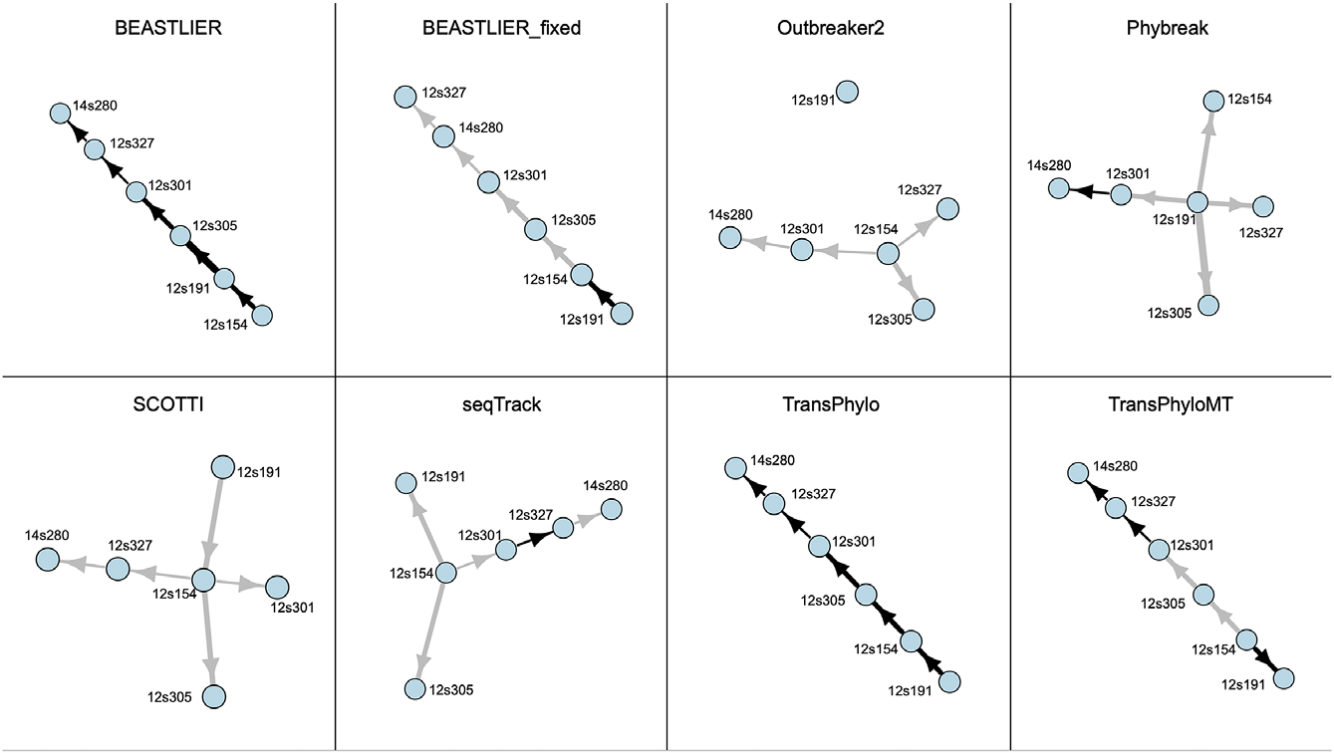
Example transmission networks predicted by each tested method for cluster MCLUST006 (*n* = 6) of *Mycobacterium tuberculosis* strains from British Columbia. Nodes represent sampled hosts and edges are the highest probability transmission link between hosts. Edge widths are weighted by the SNP distance between connected hosts, and edges are coloured black if the posterior probability of direct transmission ≥0.5 and grey if <0.5.

We observed a high degree of variation in the number of high-likelihood transmission events (posterior probability of ≥0.5) that were predicted using each method, reflecting the different underlying model algorithms and parameters. Example transmission networks produced by each method for cluster MCLUST006 (*N* = 6 cases) are shown in Figure 2.

Table 2 shows the results from each model for predicting transmission events in the 12 real-world *Mtb* clusters from BC, Canada. Overall, the number of the epidemiologically linked case–contact pairs identified by all methods was low and there was considerable variability in the number of these links that were identified within the different clusters (Supplementary Table S2). The model that predicted the highest number of transmission links supported by case–contact data was Phybreak (17/120; 14%). This was followed by TransPhylo, Outbreaker2, and BEASTLIER, all detecting 14 of the 120 case–contact pairs (12%). Considering the number of identified case–contact pairs as a percentage of the total high-likelihood transmission links predicted by each model, the highest-tested model was TransPhyloMT, with 31% of the total transmission links supported by case–contact data. This suggests that although fewer case–contact pairs were identified with this model, there were also a lower proportion of spurious links predicted. SCOTTI and BEASTLIER_fixed inferred very few high-likelihood transmission events overall, which resulted in a low number of predicted links that were validated by case–contact data.

**Table 2.** The results of predicted transmission reconstruction model for identifying transmission links in real-world *Mtb* clusters in BC that are supported by case–contact data. Bolded values are the best performing models.

Transmission reconstruction model	Number of high-likelihood transmission links (*P* ≥ 0.5)	Number of high-likelihood transmission links supported by case–contact data	Percentage of case–contact links identified (*N* = 120)	Percentage of high-likelihood transmission links supported by case–contact data
BEASTLIER	320	14	12%	4%
BEASTLIER_fixed	15	2	2%	13%
Outbreaker2	133	14	12%	11%
Phybreak	142	17	**14%**	12%
SCOTTI	4	1	1%	25%
seqTrack	80	8	7%	10%
TransPhylo	81	14	12%	17%
TransPhyloMT	26	8	7%	**31%**

**Figure 3.**
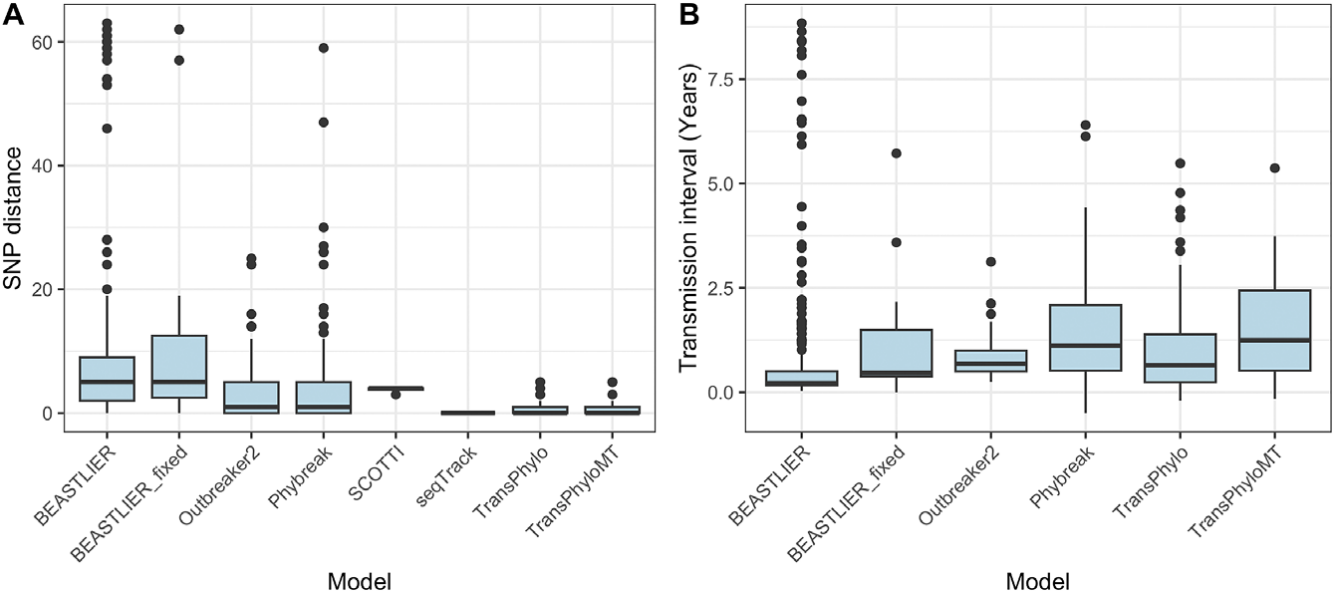
Boxplots of the transmission parameters estimated by each tested method from high-probability (*P* ≥ 0.5) transmission events between sampled *Mycobacterium tuberculosis* isolates from British Columbia. (a) The SNP distance between observed hosts, and (b) the transmission interval between infection times of observed hosts. Note that SCOTTI and seqTrack do not estimate infection times.

Posterior estimates of SNP distance and transmission interval between hosts in predicted direct transmission events revealed differences between tested approaches. The median SNP distance in high-probability transmission links between observed hosts was low for all models, ranging from zero SNPs (IQR 0–0 SNPs) in seqTrack, which only predicted transmission events between identical sequences, to five SNPs (IQR 2–12 SNPs) for BEASTLIER_fixed (Figure 3a). BEASTLIER, BEASTLIER_fixed, and Phybreak, which do not consider unsampled hosts, predicted several transmission events between relatively divergent isolates (>30 SNP differences), which is likely due to inferred direct transmission between sampled hosts when there may be unobserved cases in the real transmission chains.

Figure 3b illustrates the differences in the transmission interval between observed hosts in direct transmission events for all models that estimated an infection time for hosts. All tested models estimate that most secondary cases are within the first 2 years after donor host infection, ranging from a median interval of 0.2 years (IQR 0.2–0.5) with BEASTLIER to 1.2 years (QR 0.5–2.4 years) with TransPhyloMT. BEASTLIER predicted some high-probability transmission events between hosts where the infection time is many years, with a maximum value of 8.8 years between host infection times. While long periods between transmission events have been observed in *Mtb* due to latency of disease onset [37], these predicted transmission links with a large interval were not supported by epidemiological evidence and, again, are likely due to this model not accounting for unsampled hosts in the transmission network.

### Sensitivity analysis

To assess the impact of setting the accepted probability threshold of transmission links at *P* ≥ 0.5, we re-calculated the number of transmission links that were supported by case–contact data in the BC *Mtb* clusters at probability thresholds of *P* ≥ 0.75 and *P* ≥ 0.25. As expected, we found an increase in the number of these links that were with most models at the lower probability threshold of ≥0.25, and a decrease in the number of pairs identified at *P* ≥ 0.75 (Supplementary Figure S1A). These differences were most pronounced in SCOTTI and BEASTLIER_fixed, where lowering the probability threshold allowed for a greater number of case–contact pairs to be identified. Lowering the probability threshold also increased the overall number of transmission links that were predicted with each tested model, which in turn, increased the percentage of predicted links that were not supported by case–contact data with most approaches (Supplementary Figure S1B). Interestingly, this was not seen in seqTrack where the percentage of links that were supported by case–contact data remained similar at all thresholds, though the percentage of epidemiologically supported links increased markedly at the lower threshold. This was also seen in BEASTLIER, though approximately the same number of links were predicted with all probability thresholds with this model.

## Discussion

We present the first systematic comparison of tools for reconstructing TB transmission from WGS data in simulated and real-world *Mtb* transmission outbreaks. Our results demonstrate that the choice of tool can impact the number and accuracy of predicted transmission links. We found the accuracy of all tested models was relatively low for reconstructing transmission links using only genomic and temporal data, though some approaches can lend evidence to potential transmission between hosts and confirm the absence of transmission. As such, the results presented here can help to guide the choice of tool for *Mtb* transmission investigations and highlight potential areas of development when using genomic data to reconstruct transmission in these populations.

We found that Phybreak, Outbreaker2, and TransPhylo performed better in predicting true positive transmission links with the fewest false positive links in simulated TB outbreaks. TransPhylo and Phybreak performed best on simulations produced by their respective tools, although both performed higher than most other tools across all simulations. Notably, the specificity of all models was high, suggesting these tools may be most useful to refute transmission between hosts. To accurately assess model performance, the PPV and specificity should be taken in context with the sensitivity. In examples where few transmission links are predicted, the contribution of correctly predicted links to increasing the PPV will be higher than when many links are predicted. There were differences in model performance between the outbreaks produced using the two simulation methods that can be explained by differences in the underlying transmission networks. Simulated outbreaks with TransPhylo had more cases where a single host infected many individuals (Supplementary Figure S2). Though this is seen in real *Mtb* outbreaks, accurately reconstructing transmission in these circumstances can be difficult as descendant hosts will be genetically close and may be spuriously linked by direct transmission. Therefore, this suggests that the epidemiology of TB outbreaks can also influence the accuracy of these tools.

This study was limited by the absence of gold-standard confirmation of transmission between individuals in our real-world *Mtb* clusters. We were able to provide evidence of potential transmission between sampled hosts by including case–contact data in our analysis. These links would not fully account for all transmission between sampled hosts as contacts would be routinely missed in this form of data collection. In addition, the size of our clusters varied considerably so it was not possible to calculate meaningful statistics for our BC TB clusters that included a measure of true negatives or false positives, such as the specificity. Nonetheless, we hypothesised that the presence of epidemiological and genomic linkage between hosts increased the likelihood of these links being true transmission events.

The number of case–contact pairs identified by all methods was relatively low in our real-world TB clusters, with Phybreak achieving the highest sensitivity but only finding 17% of epidemiologically supported links. TransPhyloMT had the highest proportion of predicted transmission links that were supported by case–contact data, though over two-thirds of these links did not match with a case–contact pair. Most transmission links predicted by all models were within realistic estimates of the SNP distance and time between infection in *Mtb* transmission pairs. Most inferred transmission was between pairs separated by five or fewer SNPs and under 2 years between host infection times. While the transmission interval can be highly variable in TB, evidence suggests that most secondary transmission events occur within 2 years [34, 38], and this should be reflected in the predicted transmission. There were some transmission events predicted by BEASTLIER and Phybreak between hosts separated by many SNPs and multiple years apart, though these models do not allow for unsampled hosts and attempt to reconstruct a full transmission network between all samples.

Poor model performance in the BC data appears to be driven by the low number of case–contact pairs inferred in the largest two transmission clusters, MCLUST001 and MCLUST002 (Supplementary Table S2). These clusters were part of two TB outbreaks with complex demography that included people experiencing homelessness and poly-substance use [39, 40]. The disparity between the inferred transmission networks and the reported case–contact information may be due to difficulty in contact tracing in these settings. Removing these large clusters from the analysis increased the overall sensitivity of most models, although only 30% of the remaining case–contact pairs were identified using the model with the highest sensitivity, Phybreak. Reducing the probability threshold to accept linkage between hosts improved model sensitivity but led to a greater number of unsupported links predicted by most models. Given the low accuracy in predicting transmission demonstrated in this study, it may be valuable to provide the probability of linkage between hosts rather than a binary classifier based on a probability threshold for accepting transmission, which has been used previously in studies using these tools to gain insights in the *Mtb* transmission dynamics (e.g., [19, 21, 31]).

The best-performing models in our study differed from those identified by Firestone et al. from foot-and-mouth outbreaks [18], with Outbreaker2 and TransPhylo performing markedly better in our study. Here, we did not use any spatial or contact data to inform our transmission inference and there are key epidemiological and genetic differences between the pathogens tested. The sensitivity of TransPhylo was far lower in our results than in simulated outbreaks reported by the authors of the model, with the highest median sensitivity in our simulated outbreaks at 0.26, compared to 0.72 in the original paper. The difference in sensitivity between the analyses is likely due to the original study inferring the transmission tree from a phylogeny produced directly from the simulations, while we reconstructed the phylogeny from the output sequence data, increasing the phylogenetic uncertainty. While this study is not an exhaustive analysis of all transmission network reconstruction models, we have presented results from selected methods that have been used to reconstruct pathogen transmission and where well-documented software packages were freely available. For this reason, we were unable to run the BORIS package based on the best-performing model in Firestone et al. as there was no clear documentation supplied to apply this tool [18]. We did not fully explore the impact of varying parameter distributions on the resulting transmission inferences owing to the scale of this analysis, though testing a range of parameter choices can also be important to maximise the utility of the tested tools.

Developing computational models for transmission inference to better reflect the characteristics of disease outbreaks and incorporate more realistic epidemiological parameters may improve the prediction of transmission networks in TB. This is reflected in the results here, where the best-performing models, Phybreak, Outbreaker2, and TransPhylo were developed to account for the complex epidemiology of infectious diseases like TB, accounting for within-host evolution and incomplete sampling [15, 17, 29]. Approaches to improve TB transmission reconstruction from genomic data should include modifying existing tools to include other forms of observed variation between strains, such as small insertions and deletions (INDELs) and structural variants, as well as focusing efforts on increasing the detectable variation between *Mtb* strains using long-read sequencing and improving variant calling of minor frequency SNPs. These strategies may improve resolution in outbreaks when multiple isolates are separated by very few SNPs, which is commonplace in *Mtb* populations with low genomic diversity.

A further consideration when choosing the transmission reconstruction model is the processing complexity and runtime required, particularly for real-time surveillance of *Mtb* transmission or in settings with limited computational resources. The runtime of each approach varied considerably (Supplementary Table S3), with seqTrack computing the transmission network almost instantly for the largest BC *Mtb* cluster MCLUST002 (*N* = 74 cases). Tools that employ a Bayesian framework must typically run over millions of MCMC iterations for convergence and thus took considerably longer.

In conclusion, we have systematically compared six tools for reconstructing transmission using genomic data to assess their utility for TB transmission analysis. While there were limitations in the accuracy of the transmission links predicted by all models, we found that Phybreak, Outbreaker2, and TransPhylo identified the highest number of true links in outbreak simulation. Moreover, almost half of the high-probability transmission events predicted using these models were true transmission links and all models had a high specificity for refuting transmission between unlinked hosts. These approaches could be applied to gain some insights into *Mtb* transmission dynamics using sequence data in settings with limited contact network information. These findings can improve investigations into *Mtb* outbreaks and transmission dynamics and highlight further areas of research to advance methods for transmission network reconstruction.

## Data Availability

Whole genome sequence data included in this study are deposited in the European Nucleotide Archive (ENA) Project number PRJNA413593.

## Supplementary material

For supplementary material accompanying this paper visit http://doi.org/10.1017/S0950268823000900. click here to view supplementary material
