# Supplementary material for: Comparing Mycobacterium tuberculosis transmission reconstruction models from whole genome sequence data
Source: Epidemiol Infect. 2023 Jun 9;151:e105. doi: 10.1017/S0950268823000900 (PMC10369424; doi:10.1017/S0950268823000900)
Supplement: Supplementary file 1 [file hygsup.zip › S0950268823000900sup001.docx]

Comparing Mycobacterium tuberculosis transmission reconstruction models from whole genome sequence data (Supplementary Methods)

Benjamin Sobkowiak ^1,2,*^ , Kamila Romanowski ^2,3^, Inna Sekirov ^2,4^, Jennifer L Gardy ^5^, James Johnston ^1,2^

1. Division of Respiratory Medicine, University of British Columbia, Vancouver, Canada.

2. British Columbia Centre for Disease Control, Vancouver, Canada.

3. School of Medicine, University of British Columbia, Vancouver, Canada.

4. Department of Pathology and Laboratory Medicine, University of British Columbia, Vancouver, BC, Canada.

5. Bill and Melinda Gates Foundation, Seattle, WA, USA.

* Corresponding Author: bs2259@yale.edu

**Supplementary Methods**

***Phylogenetic tree construction of clinical Mycobacterium tuberculosis (Mtb) isolates***

To construct phylogenetic trees for real-world whole genome sequence data from British Columbia, Canada, for each MIRU-VNTR cluster with either BEAST v1.10.4 [1] or BEAST2 v2.6.3 [2], a multiple sequence alignment of concatenated SNPs was used as input. Firstly, an appropriate substitution rate model was chosen for each cluster by producing a maximum likelihood tree using IQTREE [3] with 1000 bootstraps and applying the ‘ModelFinder’ algorithm [4] to find the model with the lowest ﻿Bayesian information criterion (BIC) score. We assessed the temporal signal in each cluster (the correlation between collection date and genomic distance) using TempEst [5]. Where some temporal signal was found (*R^2^ >* 0.1), a strict molecular clock was used, otherwise we used a relaxed lognormal clock prior and supplied both models with an initial prior of 1x10^-7^ with a lognormal distribution, updated through MCMC iterations. Tree building parameters were further optimised for each cluster by conducting separate preliminary runs of 10^8^ MCMC iterations whilst varying the population model (constant, exponential, and Bayesian Skyline). XML files modified manually to estimate the number of invariant sites. Results were assessed using the posterior marginal likelihood estimates and run convergence in Tracer v1.7.1 [6], discarding the 10% of trees as the burn-in. The optimal tree building parameters for each cluster, along with the posterior estimates of cluster-specific substitution rate and tree height, are shown below.

| **Cluster Name** | **Substitution model** | **Root to tip correlation** | **No. of variants** | **Population model** | **Clock rate** | **Estimated substitution Rate** |
| --- | --- | --- | --- | --- | --- | --- |
| **MCLUST001** | HKY | 0.189 | 2250 | Constant | Strict | 1.23E-07 |
| **MCLUST002** | HKY | 0.301 | 2274 | Constant | Strict | 1.01E-07 |
| **MCLUST003** | HKY | 0.422 | 548 | Constant | Strict | 1.55E-07 |
| **MCLUST004** | HKY | 0.226 | 825 | Constant | Strict | 4.21E-07 |
| **MCLUST006** | HKY | 0.120 | 904 | Constant | Strict | 9.98E-07 |
| **MCLUST007** | HKY | 0.316 | 614 | Constant | Strict | 1.18E-06 |
| **MCLUST008** | HKY | 0.417 | 1306 | EBSP | Strict | 1.44E-07 |
| **MCLUST012_1** | HKY | 0.196 | 1244 | Constant | Strict | 1.17E-07 |
| **MCLUST012_2** | HKY | 0.119 | 1244 | Constant | Strict | 1.58E-07 |
| **MCLUST052** | HKY | 0.541 | 847 | Constant | Strict | 1.83E-07 |
| **MCLUST058** | HKY | 0.102 | 1257 | Constant | Strict | 4.12E-07 |
| **MCLUST134** | HKY | 0.338 | 935 | Constant | Strict | 1.73E-07 |

**Supplementary methods table 1**. Summary SNP statistics, prior tree parameters and posterior estimated substitution rates for all MIRU-VNTR *Mtb* cluster from BC, estimated using BEAST2.

Final time-calibrated phylogenies were produced from BEAST2 runs of 10^9^ MCMC iterations with the optimised prior parameters, sampling every 10,000^th^ tree and discarding the first 10% of trees as the burn-in. Model convergence was verified by checking that all posterior parameters showed an effective sample size (ESS) of greater than 200 in Tracer (v.1.7.1) [6]. Where a single phylogenetic tree was used as input for reconstruction models, a maximum clade consensus tree was obtained using Tree Annotator v2.6.3 [2] with median node heights.

Preliminary analysis revealed that one cluster of 65 individuals, MCLUST012, was comprised of two distinct sub-clusters of 49 and 16 individuals, and thus was split for the transmission reconstruction analysis.

***Model specific parameters for tested transmission reconstruction tools***

The following are model specific methods and parameter choices for each of the tested models used to reconstruct transmission in simulated and clinical clusters of *Mycobacterium tuberculosis*.

*BEASTLIER*

BEASTLIER [7] works by modifying the input XML file used by BEAST v1.8.4 or later to estimate transmission probabilities between hosts to produce a consensus transmission network, along with the phylogenetic tree output from BEAST. Resulting transmission trees does not allow for non-sampled hosts in the inferred network. We used the posterior distribution of transmission trees to calculate the pairwise probability of transmission between all pairs in our clusters.

A python2 script generates a new input file for BEAST and requires three input files: the standard XML file produced by BEAUTi, a CSV file containing the name and date of becoming non-infectious for each host, and a CSV file containing the sequence name, host name and collection dates of each sequence. The date that a host became non-infectious was set as three months past the collection date (using collection date as a proxy for the date of treatment onset) [8], and as we had only one sequence per host, a dummy host name was supplied to avoid replication of sequence names. The input XML file was produced with BEAUTi as described in the main manuscript.

The period of infectiousness, defined as the time between a host becoming infectious and end of infectiousness, was given a gamma distribution with shape and scale parameters of 1.1 and 2.5, reflecting the BCCDC guidelines for contact investigation stating that an infectious period of at least three months prior to diagnosis is projected, though with a reasonably high active case finding in our population suggesting few cases will stay infectious for a prolonged period [9]. The period of latency, defined as the period between a host being infected and becoming infectious, was also given a gamma distribution with shape and scale parameters of 1.3 and 3.33, allowing for a variable latency period with a long-tailed distribution but with the incubation period of most cases falling with the first 6 months to two years, as is estimated in low-burden settings [10–13]. The within-host demographic model was kept retained from the model described in Hall *et al* [7]. MCMC chains were run for at least 50 million iterations or until model convergence (ESS score > 200), logging every 10,000 iterations, with a 10% burn-in. BEASTLIER analysis was also repeated with a fixed input phylogenetic tree by adapting the XML file to include the ML tree in the Newick format.

*Outbreaker2*

Outbreaker2 [14] is an update of the Outbreaker tool [15] implemented in R, which uses a Bayesian framework for reconstructing outbreaks for sequence data but now allows for modulization with user-defined models for the likelihood, priors and movement estimation. While the option to customize these components was available, we ran this analysis with the pre-defined models in Outbreaker2. The updated model allows for within-host evolution and non-sampled hosts. We supplied the tool with sequence data, as a multi-sequence alignment in FASTA format, and collection dates. Prior epidemiological parameters for the generation time distribution, colonization (sampling) time distribution, sampling proportion and mutation rate can be specified with a fixed value or updated through MCMC runs. For our analysis, the generation time distribution (gamma distribution with shape = 1.3 and rate = 0.3), sampling time distribution (gamma distribution with shape = 1.1 and rate = 0.4), and initial mutation rate estimated from each cluster’s phylogeny in BEAST2 (**Supplementary methods table 1**), updated through MCMC iterations [16,17]. We estimated up to 80% of cases would be captured in our dataset, reflecting the effective case-finding strategy in BC [10], and thus we set a sampling proportion with informative beta priors of 8 and 2, updated through MCMC runs. The model was run for 10^5^ MCMC chains, sampled every 10^3^ iterations.

*Phybreak*

Phybreak [18] is implemented in R and uses Bayesian statistics to infer both transmission and phylogenetic trees simultaneously. The tool uses sequence data, as a multi-sequence FASTA file, and collection date to produce a ‘phybreakdata’ object as input to the main algorithm, where you can supply user-defined prior parameters. We again set the generation time distribution (gamma distribution with shape = 1.3 and mean = 4.3), sampling time distribution (gamma distribution with shape = 1.1 and mean = 2.75), and initial mutation rate estimated from each cluster’s phylogeny in BEAST2 (**Supplementary methods table 1**), updated through MCMC iterations [16,17]. We set a within-host effective population size with a slope rate of 1.48 x t (time after infection), based on previous estimates [16]. The model was run for 10^5^ MCMC chains, sampled every 10^3^ iterations.

*SCOTTI*

SCOTTI [19] is available as an extension package in BEAST2 and estimates transmission probabilities between samples, along with sampling from a pool of generic non-sampled hosts (with the number specified by the user), to reconstruct transmission trees using a structed coalescent model [19]. A python script is used to create a modified XML file that can be run with BEAST2 and requires four input files: a FASTA sequence file, a CSV file of the sampling dates, a CSV file of the host names, and a CSV of infection intervals for each host. Again, in our dataset there was one sequence per host so dummy host names were used. The infection interval spans the earliest time a host is infectible to the latest time in which they are infectious. We allowed for periods of latency where an individual may be infected before the disease becomes active by setting the earliest time for each host to be able to be a recipient of transmission as three years prior to the earliest sample collection date, and the end date of infectiousness to be three months after the collection date [8].

We allowed SCOTTI to include non-sampled hosts in the resulting transmission trees by providing the maximum number of hosts (observed and unobserved) in the tree. Again, as we estimate that we have captured a high proportion of the cases in our population (70-80% of all cases), we set the maximum number of hosts as 1.5x the number of observed hosts. We also set the mutation model to be employed for each cluster (Table S1) and an estimation of the number of invariable sites per nucleotide. MCMC chains were run for at least 50 million iterations or until model convergence, logging every 10,000 iterations.

*SeqTrack*

SeqTrack [20] uses a graph approach to reconstruct transmission trees directly from sequence data, considering ancestry between all sampled hosts, not allowing for non-sampled individuals in the network. Where there is equal likelihood of transmission between multiple hosts, these conflicts are resolved by finding the most parsimonious link when likelihood of the genetic differentiation using collection dates. This tool is run in R and uses sequence data, as a multi-sequence FASTA file, and collection dates. We also supplied a mutation rate (estimated from each cluster’s phylogeny in BEAST2 (**Supplementary methods table 1**)), and sequence length (4,411,532bp).

*TransPhylo*

TransPhylo [16] is a package in R that employs a stochastic branching process in a Bayesian framework for reconstructing transmission networks via a Monte Carlo Markov chain from genomic data, notably allowing for within-host evolution and incomplete sampling of the population by including non-sampled hosts into resulting networks. This approach requires a timed phylogenetic tree as input, with sampled hosts corresponding to the tips. Prior epidemiological parameters for the generation time distribution, sampling time distribution, sampling proportion and within-host effective coalescence rate can be specified and either given a fixed value or be updated through MCMC runs. For our analysis, the generation time distribution (gamma distribution with shape = 1.3 and rate = 0.3), sampling time distribution (gamma distribution with shape = 1.1 and rate = 0.4), and a fixed within-host effective coalescence rate (100/365) were chosen based on a previous analysis of the a transmission cluster in this study population [16]. We again estimated up to 80% of cases would be captured in our dataset and thus we set an initial sampling proportion of 0.8 with informative beta priors of 8 and 2, updated through MCMC runs. The algorithm was run for 10^5^ MCMC chains, with the end date of the clusters set as three years past the latest sample collection date in the cluster to allow for some uncaptured transmission from later samples to non-sampled hosts to be potentially resolved.

Additionally, there is an extension to the original TransPhylo package that aims to improve the inferred transmission network by accounting for some uncertainty in the input phylogenetic tree by instead running simultaneous inferences with the input trees drawn from a random distribution of posterior trees produced by BEAST2 [21]. Different model parameters can be shared across runs, which allows for better mixing of the MCMC iterations. To run this method, which we refer to as TransPhyloMT, we selected 50 input phylogenetic trees drawn randomly from the posterior selection of inferred phylogenies (after a 10% burn-in) produced by BEAST2 for each cluster using a custom bash script. All model parameters were kept the same as the single tree implementation, with parameter sharing, and transmission probabilities calculated as the mean probability of host-host transmission across the 50 runs.

**Simulated Mtb clusters**

*Phybreak ‘sim.outbreak’* *clusters*

Ten clusters were simulated using the ‘sim.outbreak’ function in Phybreak. We chose the same gamma generation and sampling time distributions used as the prior estimates in transmission reconstruction models (gamma distribution with shape = 1.3 and rate = 0.3 and gamma distribution with shape = 1.1 and rate = 0.4 respectively). The sequence length was set as length of the H37Rv Mtb reference stain at 4.4M base pairs and we allowed for within-host evolution at the rate of 1.48 as previously estimated [12,13,16] and a mutation rate of 0.75 SNPs per genome per year, which is in line with previous estimates of the Mtb mutation rate [17] and is the median mutation rate estimated in our BC clusters by BEAST2 analysis (**Supplementary methods table 1**). We simulated clusters with an observed host number of 50 and then sampled 6 years either side of the median sampling date to simulate an ongoing outbreak that was active before the first observed host. To allow for the presence of non-sampled hosts within clusters, we randomly down-sampled the observed hosts within each cluster by 20% to simulate incomplete sampling, which resulted in a final sample number in clusters of between 28 and 40 observed hosts. In real-world outbreaks the likelihood of an infected host being sampled can relate to multiple factors including location, smear-status, severity of symptoms, and contact with previously diagnosed TB cases, we chose to randomly chose hosts to be sampled as an un-biased method to allow for incomplete sampling of the simulated outbreaks.

*TransPhylo ‘simulateOutbreak’ and SeqGen clusters*

Ten clusters were simulated in a two-stage process, starting with by inferring TB outbreaks and extracting the underlying transmission network and phylogenetic tree using the ‘simulateOutbreak’ function in TransPhylo [16]. Again, the same gamma generation and sampling time distributions used in transmission reconstruction models (gamma distribution with shape = 1.3 and rate = 0.3 and gamma distribution with shape = 1.1 and rate = 0.4 respectively) were used to simulate TB outbreaks. The sampling proportion was initially set to 0.8 and the final sampled outbreak size randomly chosen between 30 and 40 sampled hosts. A fixed within-host effective coalescence rate of 100/365 and timespan of nine years was used.

The resulting transmission network was used to record known transmission between hosts and the direction of transmission. The resulting phylogenetic tree was as input to SeqGen [22], which simulates mutations along a phylogeny using a Monte Carlo approach and outputs simulated genomes. First, branch lengths were rescaled from years to substitutions/genome/year by multiplying edge branch by the substitution rate of 1.13e^-7^. These trees were then used as input to SeqGen along with specifying the genome length and estimates of nucleotide proportions, which were estimated from the H37Rv reference strain. The alignment files produced by SeqGen were then used as the input to the tested methods.

**Supplementary references**

1. Drummond AJ, Suchard MA, Xie D, Rambaut A. Bayesian phylogenetics with BEAUti and the BEAST 1.7. Molecular Biology and Evolution. 2012;29:1969–73.

2. Bouckaert R, Vaughan TG, Barido-Sottani J, Duchêne S, Fourment M, Gavryushkina A, et al. BEAST 2.5: An advanced software platform for Bayesian evolutionary analysis. PLoS Computational Biology. 2019;15:1–28.

3. Minh BQ, Schmidt HA, Chernomor O, Schrempf D, Woodhams MD, Von Haeseler A, et al. IQ-TREE 2: New Models and Efficient Methods for Phylogenetic Inference in the Genomic Era. Molecular Biology and Evolution.. 2020;37:1530–4.

4. Kalyaanamoorthy S, Minh BQ, Wong TKF, Von Haeseler A, Jermiin LS. ModelFinder: Fast model selection for accurate phylogenetic estimates. Nature Methods. 2017;14:587–9.

5. Rambaut A, Lam TT, Carvalho LM, Pybus OG. Exploring the temporal structure of heterochronous sequences using TempEst (formerly Path-O-Gen). Virus Evolution. 2016;2:1–7.

6. Rambaut A, Drummond AJ, Xie D, Baele G, Suchard MA. Posterior summarization in Bayesian phylogenetics using Tracer 1.7. Systems Biology. 2018;67:901–4.

7. Hall M, Woolhouse M, Rambaut A. Epidemic Reconstruction in a Phylogenetics Framework: Transmission Trees as Partitions of the Node Set. PLoS Computational Biology. 2015;11:1–36.

8. Fitzwater SP, Caviedes L, Gilman RH, Coronel J, LaChira D, Salazar C, et al. Prolonged Infectiousness of Tuberculosis Patients in a Directly Observed Therapy Short‐Course Program with Standardized Therapy. Clinical Infectious Diseases. 2010;51:371–8.

9. BC Centre for Disease Control. Communicable Disease Control Manual Chapter 4 : Tuberculosis Contact Investigation October 2019. Communicable Disease Control mannual. 2019.

10. Romanowski K, Sobkowiak B, Guthrie JL, Cook VJ, Gardy JL, Johnston JC. Using Whole Genome Sequencing to Determine the Timing of Secondary Tuberculosis in British Columbia, Canada. Clinical Infectious Diseases. 2020;50:1052–63.

11. Behr MA, Edelstein PH, Ramakrishnan L. Revisiting the timetable of tuberculosis. BMJ. 2018;362:1–10.

12. Xu Y, Stockdale JE, Naidu V, Hatherell H, Stimson J, Stagg HR, et al. Transmission analysis of a large tuberculosis outbreak in London: a mathematical modelling study using genomic data. Microbial Genomics. 2020;6.

13. Ayabina D, Ronning JO, Alfsnes K, Debech N, Brynildsrud OB, Arnesen T, et al. Genome-based transmission modelling separates imported tuberculosis from recent transmission within an immigrant population. Microbial Genomics. 2018;4:1–13.

14. Campbell F, Didelot X, Fitzjohn R, Ferguson N, Cori A, Jombart T. outbreaker2: A modular platform for outbreak reconstruction. BMC Bioinformatics. 2018;19.

15. Jombart T, Cori A, Didelot X, Cauchemez S, Fraser C, Ferguson N. Bayesian Reconstruction of Disease Outbreaks by Combining Epidemiologic and Genomic Data. PLoS Computational Biology. 2014;10.

16. Didelot X, Fraser C, Gardy J, Colijn C. Genomic infectious disease epidemiology in partially sampled and ongoing outbreaks. Molecular Biology and Evolution. 2017;34:997–1007.

17. Ford CB, Shah RR, Maeda MK, Gagneux S, Murray MB, Cohen T, et al. Mycobacterium tuberculosis mutation rate estimates from different lineages predict substantial differences in the emergence of drug-resistant tuberculosis. Nature Genetics. 2013;45:784–90.

18. Klinkenberg D, Backer JA, Didelot X, Colijn C, Wallinga J. Simultaneous inference of phylogenetic and transmission trees in infectious disease outbreaks. PLoS Computational Biology. 2017.

19. De Maio N, Wu CH, Wilson DJ. SCOTTI: Efficient Reconstruction of Transmission within Outbreaks with the Structured Coalescent. PLoS Computational Biology. 2016;12:1–23.

20. Jombart T, Eggo RM, Dodd PJ, Balloux F. Reconstructing disease outbreaks from genetic data: A graph approach. Heredity. 2011;106:383–90.

21. Xu Y, Cancino-Munoz I, Torres-Puente M, Villamayor LM, Borrás R, Borrás-Máñez M, et al. High-resolution mapping of tuberculosis transmission: Whole genome sequencing and phylogenetic modelling of a cohort from Valencia Region, Spain. PLoS Medicine. 2019;16:1–20.

22. Rambaut A, Grassly NC. Seq-gen: An application for the monte carlo simulation of dna sequence evolution along phylogenetic trees. Bioinformatics. 1997;13:235–8.
