# Supplementary material for: Comparing Mycobacterium tuberculosis transmission reconstruction models from whole genome sequence data
Source: Epidemiol Infect. 2023 Jun 9;151:e105. doi: 10.1017/S0950268823000900 (PMC10369424; doi:10.1017/S0950268823000900)
Supplement: Supplementary file 1 [file hygsup.zip › S0950268823000900sup002.docx]

**Supporting information**

**S1 and S2 table**. XLSX file showing the full results for predicted transmission events by each model in simulated and real-world *Mycobacterium tuberculosis* transmission clusters.

**S3 Table.** Table showing the run times for each model on real-world *Mycobacterium tuberculosis* transmission cluster MCLUST002.

**S1 Figure.** (A) The percentage of case-contact pairs identified and (B) the percentage of inferred transmission links supported by case-contact pairs by each tested model on real-world *Mycobacterium tuberculosis* transmission clusters with varying probability thresholds.

**S2 Figure.** (A) The SNP distance between known transmission pairs and (B) the secondary offspring distribution of all hosts in simulated TB outbreaks.
